# Supplementary material for: Risk factors associated with high altitude sickness among travelers: A case control study in Himalaya district of Nepal
Source: PLOS Glob Public Health. 2025 Feb 7;5(2):e0004241. doi: 10.1371/journal.pgph.0004241 (PMC11805385; doi:10.1371/journal.pgph.0004241)
Supplement: Attachment — Submitted filename: PGPH-D-24-00773_reviewer_comments.docx [file pgph.0004241.s002.docx]

This case-control study assesses the risk factors associated with altitude illness and highlights the importance of various factors in relation to the development of altitude illness.

INTRODUCTION

Line 61 – Need to define HACE and HAPE before using abbreviations.

Lines 107-108 the sentence ‘However, rapidly ascending from lower

107 elevations to such hypobaric hypoxia environment, thereby increasing the risk for developing high altitude illness for many travelers’ – not sure this is an appropriate statement for the methods section. You already state/imply this in the introduction in lines 84-86 anyways.

Line 115: ‘Irrespective of residence altitude’ – this implies that they could be acclimatized? Or high altitude resident? And in relation to this, what if participants were matched that resided at different altitudes?

Lines 125: LLS is a symptom severity questionnaire that is highly subjective rather than a clinical evaluation. Be careful with the wording here.

Lines 126 – 128: Sleep has been removed from the most current LLS scoring criteria (2018). In this instance you must specifiy reference the use of the old version of the questionnaire which includes sleep.

Lines 130: Again you’ve referenced the newest version of the LLS, however, this particular version has removed sleep from the scoring system.

Line 155: ‘standard questionnaire’ – is this a healthy history questionnaire? what information was this gathering? General demographics?

Line 162 – Consistency checks? Were these checked by 2 reviewers? Or were and certain percentage of the data randomly re-checked with the hard data sheets?

Ethical considerations – perhaps this is a journal specific thing but typically this goes at the beginning of the methods section. Does the NHRC provide an ethical review number, worth referencing this specifically.

It is somewhat unclear how ‘awareness’ was assessed and what exactly ‘awareness’ was for? Awareness that high-altitude illnesses exist, and knowing what the symptoms are? Or was it awareness of medications used for altitude illness? Or both. Authors should be more clear about the assessment of ‘awareness’ in the methods section.

RESULTS

Lines 212-214: Unsure why worth pointing out an insignificant association especially given it is already presented in the Table 3. Avoid duplicating results in table and text.

Also unclear to reader why there are adjusted and unadjusted OR presented? Is this for if they were calculated as a singular variable and then adjusted for multivariate analysis?

DISCUSSION

Line 261- You already define and use the abbreviation for AMS so you need not to do this again in the discussion.

Line 269 – acetazolamide in parenthesis should be changed to (i.e., acetazolamide). I also believe through the paper that acetazolamide is capitalised , however, as a generic drug, generally, this is not capitalized where as, Diamox (the brand name) would be capitalised.

General thought for future directions - Were individuals with history of altitude sickness also more likely to take medication to prevent sickness? And were those with previous sickness also the individuals who had the most ‘awareness’? Perhaps this is beyond the scope but it would be interesting to know whether ‘awareness’ is essentially spawned from people getting sick or whether they are actually being educated about altitude sickness. Perhaps an important thing to know moving forward in terms of how to improve the amount of people getting sick!

The most important thing to highlight is how is this study contributes anything different to existing studies in regards to further understanding of risk factors related to sickness. After all you are able to state the primary and significant risk factors in the introduction already, so it will me important to highlight how this study contributes something additional/provide something different to the existing literature. For example, is it the only (or one of very limited) case-control study of it’s kind? Etc.
