# Supplementary material for: Risk factors associated with high altitude sickness among travelers: A case control study in Himalaya district of Nepal
Source: PLOS Glob Public Health. 2025 Feb 7;5(2):e0004241. doi: 10.1371/journal.pgph.0004241 (PMC11805385; doi:10.1371/journal.pgph.0004241)
Supplement: Attachment — Submitted filename: Response to Reviewer.docx [file pgph.0004241.s004.docx]

**Rebuttal letter**

**Response to reviewers**

We sincerely thank the editor and reviewers for their time and effort in evaluating our manuscript. Below, we provide detailed responses to each comment raised. The review decision was retrieved from the editorial communication via email. We have used the same document to structure our responses.

**Editor’s comments**

1. Please include a complete copy of PLOS’ questionnaire on inclusivity in global research in your revised manuscript. Our policy for research in this area aims to improve transparency in the reporting of research performed outside of researchers’ own country or community. The policy applies to researchers who have travelled to a different country to conduct research, research with Indigenous populations or their lands, and research on cultural artefacts. The questionnaire can also be requested at the journal’s discretion for any other submissions, even if these conditions are not met. Please find more information on the policy and a link to download a blank copy of the questionnaire here: https://journals.plos.org/globalpublichealth/s/best-practices-in-research-reporting. Please upload a completed version of your questionnaire as Supporting Information when you resubmit your manuscript.

**Response:** Thank you for the comment. We have added the file.

2. Thank you for uploading your study's underlying data set. Unfortunately, the repository you have noted in your Data Availability statement does not qualify as an acceptable data repository according to PLOS's standards.

At this time, please upload the minimal data set necessary to replicate your study's findings to a stable, public repository (such as figshare or Dryad) and provide us with the relevant URLs, DOIs, or accession numbers that may be used to access these data. For a list of recommended repositories and additional information on PLOS standards for data deposition, please see <https://journals.plos.org/plosone/s/recommended-repositories>.

**Response:** Thank you for the comment. We have uploaded the data set in the recommended repository. The link is: [**dx.doi.org/10.6084/m9.figshare.27105400**](https://dx.doi.org/10.6084/m9.figshare.27105400)

3. Please review your reference list to ensure that it is complete and correct. If you have cited papers that have been retracted, please include the rationale for doing so in the manuscript text, or remove these references and replace them with relevant current references. Any changes to the reference list should be mentioned in the rebuttal letter that accompanies your revised manuscript. If you need to cite a retracted article, indicate the article’s retracted status in the References list and also include a citation and full reference for the retraction notice.

**Response:** We have reviewed the reference lists and they are complete and correct. We have formatted the reference numbers inserted at the respective parts of the manuscript to ensure they now appear before the full stop, in accordance with proper citation guidelines.

**Additional Edits:** We have modified (line 12) one of the author’s (TPA) affiliation due to recent change in the name of the organization.

**Reviewers’ Comments (Review comments to the author)**

INTRODUCTION

Line 61 – Need to define HACE and HAPE before using abbreviations.

**Response**: Thank you for the comment. We have edited as per your feedback.

Lines 107-108 the sentence ‘However, rapidly ascending from lower

107 elevations to such hypobaric hypoxia environment, thereby increasing the risk for developing high altitude illness for many travelers’ – not sure this is an appropriate statement for the methods section. You already state/imply this in the introduction in lines 84-86 anyways.

**Response**: Thank you for the comment. We have removed the statement from the methods section.

Line 115: ‘Irrespective of residence altitude’ – this implies that they could be acclimatized? Or high altitude resident? And in relation to this, what if participants were matched that resided at different altitudes?

**Response**: Thank you for the comment. All the cases included in our study were travelers who developed altitude sickness after exposure to high altitudes. None were long-term high-altitude residents, and all resided below the altitude sickness threshold (2500m) prior to their exposure. Therefore, acclimatization or residence at high altitudes was not a factor, and we did not match participants based on place of residence.

Lines 125: LLS is a symptom severity questionnaire that is highly subjective rather than a clinical evaluation. Be careful with the wording here.

**Response**: Thank you for the comment. We initially used the term 'clinically' to indicate that no diagnostic investigations were used, which led to some misinterpretation. We have removed the term.

Lines 126 – 128: Sleep has been removed from the most current LLS scoring criteria (2018). In this instance you must specifiy reference the use of the old version of the questionnaire which includes sleep.

**Response**: Thank you for the comment. After reviewing the questionnaire and the patient's emergency card, we used the latest (2018) LLS scoring criteria. However, we mistakenly referred to the older version in the proposal, which was subsequently carried over into the manuscript. We have now corrected this in the manuscript.

Lines 130: Again you’ve referenced the newest version of the LLS, however, this particular version has removed sleep from the scoring system.

**Response:** Thank you for the comment. We have addressed this in the comment just above.

Line 155: ‘standard questionnaire’ – is this a healthy history questionnaire? what information was this gathering? General demographics?

**Response**: Thank you for the comment. It may have led to confusion. Therefore, the line was removed from the manuscript because the preceding sentence already described the questionnaire.

Line 162 – Consistency checks? Were these checked by 2 reviewers? Or were and certain percentage of the data randomly re-checked with the hard data sheets?

**Response:** Thank you for the comment. Consistency here refers to if there was duplication in responses. Yes, the team members [principal author and TPA] compared few sample of hard-data sheets with the entered data.

Ethical considerations – perhaps this is a journal specific thing but typically this goes at the beginning of the methods section. Does the NHRC provide an ethical review number, worth referencing this specifically.

**Response**: Thank you for the comment. We have included this at the last according to the journal’s guidelines. Yes, NHRC has provided us with an ethical review number which was mentioned in a separate document. We have also added the number to this section.

It is somewhat unclear how ‘awareness’ was assessed and what exactly ‘awareness’ was for? Awareness that high-altitude illnesses exist, and knowing what the symptoms are? Or was it awareness of medications used for altitude illness? Or both. Authors should be more clear about the assessment of ‘awareness’ in the methods section.

**Response:** Thank you for the comment. We were unable to find a validated scale to assess awareness of high altitude sickness. Therefore, we only asked participants if they had a general understanding of high altitude sickness, focusing mainly on its symptoms and who is at risk of developing it. We did not inquire about treatment or preventive measures. We have edited and mentioned this in line 140.

RESULTS

Lines 212-214: Unsure why worth pointing out an insignificant association especially given it is already presented in the Table 3. Avoid duplicating results in table and text.

Also unclear to reader why there are adjusted and unadjusted OR presented? Is this for if they were calculated as a singular variable and then adjusted for multivariate analysis?

**Response:** Thank you for the comment. Unadjusted OR was calculated by regressing one independent variable with a dependent variable whereas adjusted OR was calculated regressing all independent variables [showing significant relationship or having p-value less than 0.25 in unadjusted analysis] with dependent variable to remove confounding associated if any.

DISCUSSION

Line 261- You already define and use the abbreviation for AMS so you need not to do this again in the discussion.

**Response:** Thank you for the comment. We have edited accordingly.

Line 269 – acetazolamide in parenthesis should be changed to (i.e., acetazolamide). I also believe through the paper that acetazolamide is capitalised , however, as a generic drug, generally, this is not capitalized where as, Diamox (the brand name) would be capitalised.

**Response:** Thank you for the comment. We have corrected accordingly.

General thought for future directions - Were individuals with history of altitude sickness also more likely to take medication to prevent sickness? And were those with previous sickness also the individuals who had the most ‘awareness’? Perhaps this is beyond the scope but it would be interesting to know whether ‘awareness’ is essentially spawned from people getting sick or whether they are actually being educated about altitude sickness. Perhaps an important thing to know moving forward in terms of how to improve the number of people getting sick!

The most important thing to highlight is how this study contributes anything different to existing studies in regard to further understanding of risk factors related to sickness. After all you can state the primary and significant risk factors in the introduction already, so it will be important to highlight how this study contributes something additional/provide something different to the existing literature. For example, is it the only (or one of very limited) case-control study of its kind? Etc.

**Response:** Thank you for your comments and thoughts. Further study might be needed, especially focusing on the behavioral part you mentioned. We explored the lack of awareness on high-altitude sickness as a risk factor. Your suggestions about whether individuals with a history of altitude sickness are more likely to take preventive medication or have greater awareness raises an important point. While our study didn’t focus on this, future research could delve into whether awareness stems from personal experiences of sickness or from educational efforts. This insight could be valuable for improving prevention strategies.

This study adds something new to the understanding of risk factors for high-altitude sickness by being one of the few case-control studies on this topic. While previous research has identified key risk factors, it may be one of the few studies to explore how awareness and past experiences with sickness affect prevention. In the discussion section (line 275-278), we have mentioned how this study contributes differently to the existing literature.
