# Supplementary material for: Risk factors associated with high altitude sickness among travelers: A case control study in Himalaya district of Nepal
Source: PLOS Glob Public Health. 2025 Feb 7;5(2):e0004241. doi: 10.1371/journal.pgph.0004241 (PMC11805385; doi:10.1371/journal.pgph.0004241)
Supplement: Attachment — Submitted filename: Response_to_Reviewer_auresp_2.docx [file pgph.0004241.s006.docx]

**Rebuttal letter**

**Response to reviewers**

We sincerely thank the editor and reviewers for their time and effort in evaluating our manuscript. Below, we provide detailed responses to each comment raised. The review decision was retrieved from the editorial communication via email. We have used the same document to structure our responses.

**Reviewer #2 Comments (Review comments to the author)**

The manuscript addresses a highly relevant topic concerning the risk factors associated with high-altitude sickness (AMS, HAPE, HACE) among travelers to the Mustang district of Nepal. The growing popularity of adventure tourism and trekking in high-altitude regions has made this topic particularly timely and significant. The authors have approached this problem with a well-defined case-control study, which is an appropriate design for exploring associations between exposure factors and altitude sickness. Additionally, the emphasis on a specific region, Mustang, known for its significant trekking activity, adds valuable context and geographical specificity to the findings, increasing their relevance for both public health practitioners and tourism policymakers.

The manuscript's major strengths include its clear research question, the use of a well-matched case-control design (1:1 age-sex matching), and the rigorous use of logistic regression to determine associations between multiple potential risk factors and the development of altitude sickness. The statistical analysis is performed appropriately, and the authors provide a detailed discussion of the results, incorporating evidence from previous studies to contextualize their findings.

The discussion highlights the importance of ascent type, previous altitude sickness history, and prophylactic medication intake (acetazolamide) as significant risk factors for developing altitude sickness. These findings contribute to the broader understanding of risk factors that can be managed or mitigated through preventive measures, making the manuscript of potential utility for travel planners, public health experts, and individual adventurers.

While the manuscript presents several strong points, it also contains some limitations that need to be addressed for it to achieve its full potential. Firstly, the sample size is relatively small (63 cases and 63 controls). Although the case-control design helps address power limitations, the small sample size may affect the generalizability of the findings. Future studies with a larger cohort would further validate the associations observed here.

The authors used the Lake Louise Score (LLS) for diagnosing AMS, HAPE, and HACE. However, the LLS is a subjective assessment tool, and its use as the sole method for clinical evaluation should be carefully framed to avoid overstating its diagnostic power. The term "clinically evaluated" should be revised, as LLS is a symptom score rather than a diagnostic method. Moreover, some important confounders, such as physical fitness level and genetic predisposition, were not accounted for, which could limit the interpretation of the results.

Another issue lies in the presentation of some results and methodological descriptions. Certain sections, such as the criteria for control selection and sample size calculation, require more clarity. Additionally, there are redundant statements in the methods and introduction sections, which should be streamlined for a more concise presentation. The suggestions provided in the table below offer specific guidance for improving these aspects.

Detailed suggested edits:

Abstract: Line 21: "High elevation adventure is a traveler’s choice. However, it has many health repercussions like altitude sickness." "High elevation adventures are popular among travelers; however, they carry significant health risks, such as altitude sickness."

Introduction: Line 61: "AMS, HACE, or HAPE, ranging from mild discomfort to life-threatening emergencies" "Acute Mountain Sickness (AMS), High Altitude Cerebral Edema (HACE), or High-Altitude Pulmonary Edema (HAPE), ranging from mild discomfort to life-threatening emergencies.”

Introduction: Line 67: "AMS symptoms like headache, nausea/vomiting, fatigue, dizziness, or difficulty sleeping usually appear 6-12 hours post high-altitude arrival, lasting 1-3 days; untreated AMS can lead to HACE, with altered mental status and potential coma or death within 24 hours." "Symptoms of AMS, such as headache, nausea/vomiting, fatigue, dizziness, or difficulty sleeping, usually appear 6-12 hours after reaching high altitude and last 1-3 days. If untreated, AMS can progress to HACE, potentially leading to coma or death within 24 hours."

Introduction: Line 73: "Factors like age, obesity, gender, fitness level, and substance use in altitude sickness risk among mountaineers remain uncertain." "The influence of factors such as age, obesity, gender, fitness level, and substance use on the risk of altitude sickness among mountaineers remains uncertain."

Methods: Lines 107-108: "However, rapidly ascending from lower elevations to such hypobaric hypoxia environment, thereby increasing the risk for developing high-altitude illness for many travelers." Remove this statement, as it repeats information already provided in the introduction.

Methods: Line 115: "Irrespective of residence altitude" Clarify that participants were not acclimatized individuals. Suggestion: "All participants resided below 2500m before exposure to high altitude, ensuring that acclimatization was not a confounding factor."

Methods: Line 119: "A participant was eligible to be included as a control i.e. non-Altitude Sickness patient, if he/she, irrespective of place of residence, is patients aged 18 years or older presenting in emergency services department without AMS/HACE/HAPE after his/her exposure to high altitude." "A participant was eligible as a control if they were aged 18 years or older, presented to the emergency department without AMS/HACE/HAPE after high-altitude exposure, and had no history of altitude sickness."

Methods: Line 125: "Subjects were clinically evaluated for AMS using the LLS, a questionnaire that rates the severity of 4-5 symptoms of AMS." Replace "clinically evaluated" with "evaluated using the LLS questionnaire," as the LLS is subjective rather than a clinical diagnosis

Methods: Line 140: "Slow ascent was defined as an elevation gain of no more than 600 meters per night (above 3000m), reflecting approach for safe acclimatization to altitudes exceeding 3000 meters." "Slow ascent was defined as an elevation gain of no more than 600 meters per night above 3000m, reflecting a safe acclimatization approach for high altitudes."

Results: Line 182: "The study group consisted most of individuals of Nepalese nationality (75%). Majority of participants were classified as slow ascenders (65%)." "Most participants in the study were of Nepalese nationality (75%). The majority (65%) were classified as slow ascenders."

Results: Line 195: "Among the cases, majority (67%) had Acute Mountain Sickness (AMS) while others presented with High Altitude Pulmonary Edema (HAPE)(20%) and High Altitude Cerebral Edema (HACE)(13%)." "Among the cases, 67% had Acute Mountain Sickness (AMS), while 20% had High Altitude Pulmonary Edema (HAPE), and 13% had High Altitude Cerebral Edema (HACE)."

Discussion: Line 233: "Rapid ascenders demonstrated a significantly higher likelihood of experiencing altitude sickness compared to their slow ascending counterparts." "Rapid ascenders were significantly more likely to experience altitude sickness compared to slow ascenders."

Conclusion: Line 282: "Our case-control study contributes to the understanding of risk factors for altitude sickness, emphasizing the significance of ascent speed, previous history, and prophylactic measures." Consider highlighting the implications for travel planning and tourism management: "These findings emphasize the need for careful planning of ascent rates and the importance of preventive measures for travelers."

In conclusion, the manuscript presents a valuable investigation into the risk factors associated with altitude sickness among travelers in Mustang, Nepal. The use of a case-control study design and logistic regression analysis provides a solid foundation for identifying important risk factors, such as ascent speed, previous history of altitude sickness, and use of prophylactic medication. However, limitations regarding sample size, subjective diagnostic tools, and some confounders need to be addressed to strengthen the study's reliability and generalizability. I recommend minor revisions to address these concerns, improve clarity, and add supporting references where necessary. With these adjustments, the manuscript would be suitable for publication in its current journal.

**Response to the comments**

Comment: While the manuscript presents several strong points, it also contains some limitations that need to be addressed for it to achieve its full potential. Firstly, the sample size is relatively small (63 cases and 63 controls). Although the case-control design helps address power limitations, the small sample size may affect the generalizability of the findings. Future studies with a larger cohort would further validate the associations observed here.

**Response**: Thank you for the comments. We acknowledge the limitation of the small sample size (63 cases and 63 controls) and its potential impact on generalizability. So we encourage further studies in similar settings in our discussion section.

Comment: The authors used the Lake Louise Score (LLS) for diagnosing AMS, HAPE, and HACE. However, the LLS is a subjective assessment tool, and its use as the sole method for clinical evaluation should be carefully framed to avoid overstating its diagnostic power. The term "clinically evaluated" should be revised, as LLS is a symptom score rather than a diagnostic method. Moreover, some important confounders, such as physical fitness level and genetic predisposition, were not accounted for, which could limit the interpretation of the results

**Response:** Thank you for the comments.

Another issue lies in the presentation of some results and methodological descriptions. Certain sections, such as the criteria for control selection and sample size calculation, require more clarity. Additionally, there are redundant statements in the methods and introduction sections, which should be streamlined for a more concise presentation. The suggestions provided in the table below offer specific guidance for improving these aspects.

Detailed Suggested Edits

ABSTRACT

Line 21- : "High elevation adventure is a traveler’s choice. However, it has many health repercussions like altitude sickness." "High elevation adventures are popular among travelers; however, they carry significant health risks, such as altitude sickness."

Response: Thank you for the comment. We have edited accordingly.

INTRODUCTION

Line 61: "AMS, HACE, or HAPE, ranging from mild discomfort to life-threatening emergencies" "Acute Mountain Sickness (AMS), High Altitude Cerebral Edema (HACE), or High-Altitude Pulmonary Edema (HAPE), ranging from mild discomfort to life-threatening emergencies.”

**Response**: Thank you for the comment. We already corrected this sentence after the comments of reviewer #1.

Line 67: "AMS symptoms like headache, nausea/vomiting, fatigue, dizziness, or difficulty sleeping usually appear 6-12 hours post high-altitude arrival, lasting 1-3 days; untreated AMS can lead to HACE, with altered mental status and potential coma or death within 24 hours." "Symptoms of AMS, such as headache, nausea/vomiting, fatigue, dizziness, or difficulty sleeping, usually appear 6-12 hours after reaching high altitude and last 1-3 days. If untreated, AMS can progress to HACE, potentially leading to coma or death within 24 hours."

**Response**: Thank you for the comment. We have edited as per suggestion, but the two sentences have been merged.

Line 73: "Factors like age, obesity, gender, fitness level, and substance use in altitude sickness risk among mountaineers remain uncertain." "The influence of factors such as age, obesity, gender, fitness level, and substance use on the risk of altitude sickness among mountaineers remains uncertain

**Response**: Thank you for the comment. We have edited accordingly.

METHODS

Lines 107-108: "However, rapidly ascending from lower elevations to such hypobaric hypoxia environment, thereby increasing the risk for developing high-altitude illness for many travelers." Remove this statement, as it repeats information already provided in the introduction

**Response**: Thank you for the comment. It was already removed after the comments of reviewer #1.

Line 115: "Irrespective of residence altitude" Clarify that participants were not acclimatized individuals. Suggestion: "All participants resided below 2500m before exposure to high altitude, ensuring that acclimatization was not a confounding factor."

**Response**: Thank you for the comment. We have added the sentence.

Line 119: "A participant was eligible to be included as a control i.e. non-Altitude Sickness patient, if he/she, irrespective of place of residence, is patients aged 18 years or older presenting in emergency services department without AMS/HACE/HAPE after his/her exposure to high altitude." "A participant was eligible as a control if they were aged 18 years or older, presented to the emergency department without AMS/HACE/HAPE after high-altitude exposure, and had no history of altitude sickness”.

**Response:** Thank you for the comment. We have edited accordingly.

Line 125: "Subjects were clinically evaluated for AMS using the LLS, a questionnaire that rates the severity of 4-5 symptoms of AMS." Replace "clinically evaluated" with "evaluated using the LLS questionnaire," as the LLS is subjective rather than a clinical diagnosis

**Response**: Thank you for the comment. We already removed the term after comments of reviewer #1.

Line 140: "Slow ascent was defined as an elevation gain of no more than 600 meters per night (above 3000m), reflecting approach for safe acclimatization to altitudes exceeding 3000 meters." "Slow ascent was defined as an elevation gain of no more than 600 meters per night above 3000m, reflecting a safe acclimatization approach for high altitudes."

**Response:** Thank you for the comment. We have modified accordingly.

RESULTS

Line 182: "The study group consisted most of individuals of Nepalese nationality (75%). Majority of participants were classified as slow ascenders (65%)." "Most participants in the study were of Nepalese nationality (75%). The majority (65%) were classified as slow ascenders."

**Response:** Thank you for the comment. We have made changes accordingly.

Line 195: "Among the cases, majority (67%) had Acute Mountain Sickness (AMS) while others presented with High Altitude Pulmonary Edema (HAPE)(20%) and High Altitude Cerebral Edema (HACE)(13%)." "Among the cases, 67% had Acute Mountain Sickness (AMS), while 20% had High Altitude Pulmonary Edema (HAPE), and 13% had High Altitude Cerebral Edema (HACE)."

**Response:** Thank you for the comment. We have modified accordingly.

DISCUSSION

Line 233: "Rapid ascenders demonstrated a significantly higher likelihood of experiencing altitude sickness compared to their slow ascending counterparts." "Rapid ascenders were significantly more likely to experience altitude sickness compared to slow ascenders."

**Response:** Thank you for the comment. We have edited accordingly.

CONCLUSION

Line 282: "Our case-control study contributes to the understanding of risk factors for altitude sickness, emphasizing the significance of ascent speed, previous history, and prophylactic measures." Consider highlighting the implications for travel planning and tourism management: "These findings emphasize the need for careful planning of ascent rates and the importance of preventive measures for travelers."

**Response:** Thank you for the comment. We have added as per to your suggestions.

In conclusion, the manuscript presents a valuable investigation into the risk factors associated with altitude sickness among travelers in Mustang, Nepal. The use of a case-control study design and logistic regression analysis provides a solid foundation for identifying important risk factors, such as ascent speed, previous history of altitude sickness, and use of prophylactic medication. However, limitations regarding sample size, subjective diagnostic tools, and some confounders need to be addressed to strengthen the study's reliability and generalizability. I recommend minor revisions to address these concerns, improve clarity, and add supporting references where necessary. With these adjustments, the manuscript would be suitable for publication in its current journal.

**Response:** Thank you for your comments and thoughts. We have addressed the minor revisions as per your suggestions to improve the clarity of the methods and results section. While we acknowledge that various confounding factors could influence the outcomes, they were not directly measured in our study due to practical limitations, such as the challenges of objectively assessing physical fitness in a field setting. However, we have highlighted these as limitations in the discussion section and recognize their importance for future research.

OTHER EDITS

We have edited the contents on ABSTRACT section and some minor corrections in the manuscript content.
